# Supplementary material for: Immune checkpoint modulation enhances HIV-1 antibody induction
Source: Nat Commun. 2020 Feb 19;11:948. doi: 10.1038/s41467-020-14670-w (PMC7031230; doi:10.1038/s41467-020-14670-w)
Supplement: Supplementary file 1 — Supplementary Information [file 41467_2020_14670_MOESM1_ESM.pdf]

Supplementary Information

Bradley et al. Immune checkpoint modulation enhances HIV-1 antibody induction

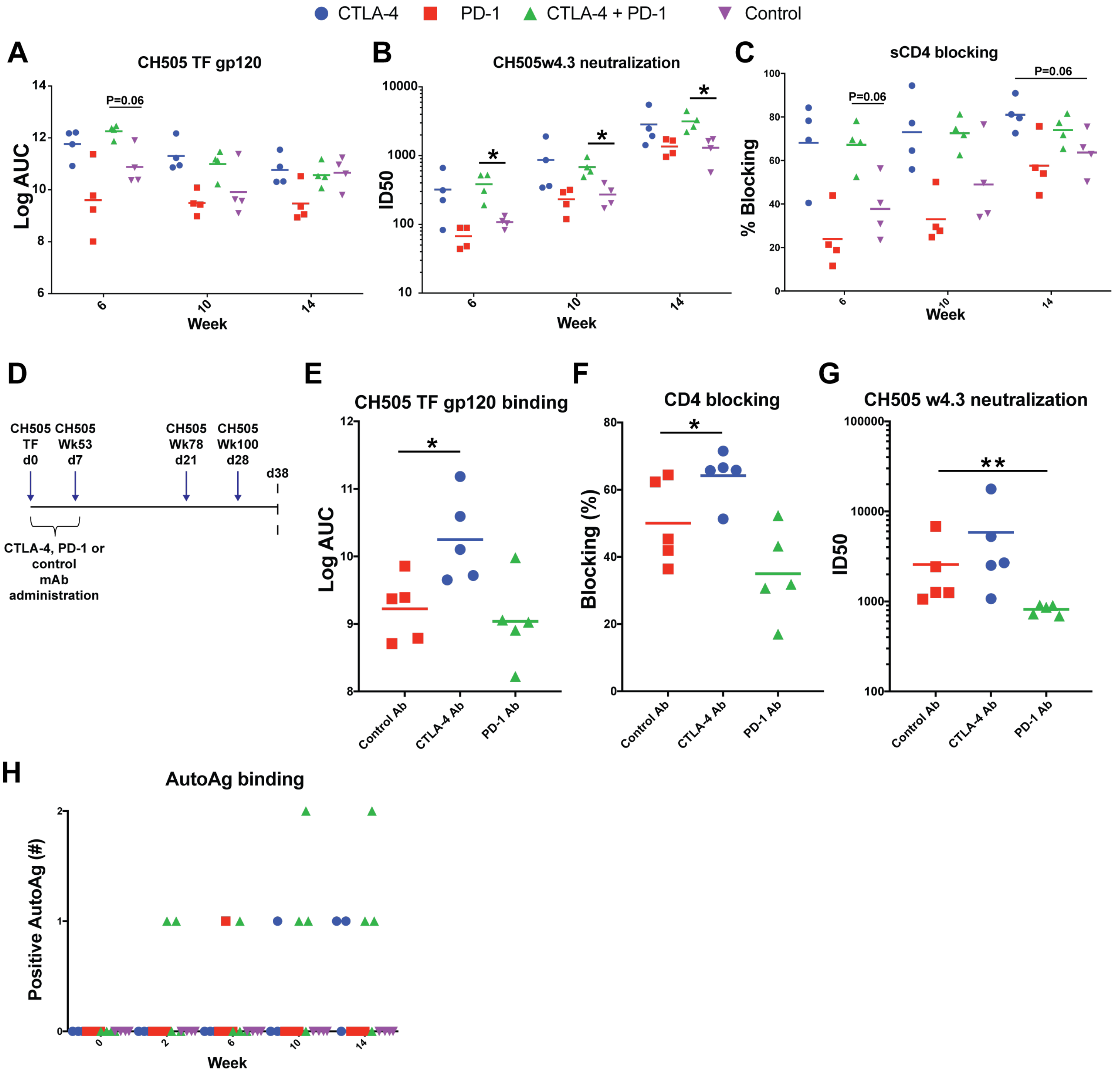
CTLA-4
PD-1
CTLA-4 + PD-1
Control
A
CH505 TF gp120
P=0.06
Log AUC
Week
B
CH505w4.3 neutralization
ID50
Week
C
sCD4 blocking
P=0.06
P=0.06
% Blocking
Week
D
CH505 TF d0
CH505 Wk53 d7
CH505 Wk78 d21
CH505 Wk100 d28
d38
CTLA-4, PD-1 or control mAb administration
E
CH505 TF gp120 binding
Log AUC
Control Ab
CTLA-4 Ab
PD-1 Ab
F
CD4 blocking
Blocking (%)
G
CH505 w4.3 neutralization
ID50
H
AutoAg binding
Positive AutoAg (#)
Week

Supplementary Figure 1. Co-administration of CTLA-4 and PD-1 blocking antibodies with HIV gp120 subunit vaccine in Cynomolgus macaques and mice. (A-D) Plasma antibody titers of **(A)** binding to CH505 TF gp120 by ELISA measured by Log area under the curve **(B)** neutralization of tier-1 autologous CH505w4.3 virus in the TZM-bl assay measured by ID50. **(C)** Blocking of soluble CD4 binding to the CH505 TF gp120 protein by ELISA. **(D)** Vaccination protocol for immunization of four groups of BALB/c mice with sequential CH505 HIV Env gp120 recombinant proteins with co-administration of CTLA-4, PD-1 or control antibodies. **(E-G)** Plasma antibody titers of **(E)** binding to CH505 TF gp120 by ELISA measured by Log area under the curve **(F)** blocking of soluble CD4 binding to the CH505 TF gp120 protein by competitive ELISA. Data shown is percent blocking and **(G)** neutralization of tier-1 autologous CH505w4.3 virus in the TZM-bl assay measured by ID50. Each dot represents a single animal and are colored based on antibody treatment group (CTLA-4, blue; PD-1, green; Control Ig, red). **(H)** Number of positive autoantigens tested in the AtheNA assay determined positive above background in the macaque study. Each dot represents a single animal and are colored based on antibody treatment group (CTLA-4, blue; PD-1, red; CTLA-4 + PD-1, green; Control CH65, purple). *$P<0.05$, **$P<0.01$, Wilcoxon-Mann-Whitney. Source data are provided as a Source Data file.

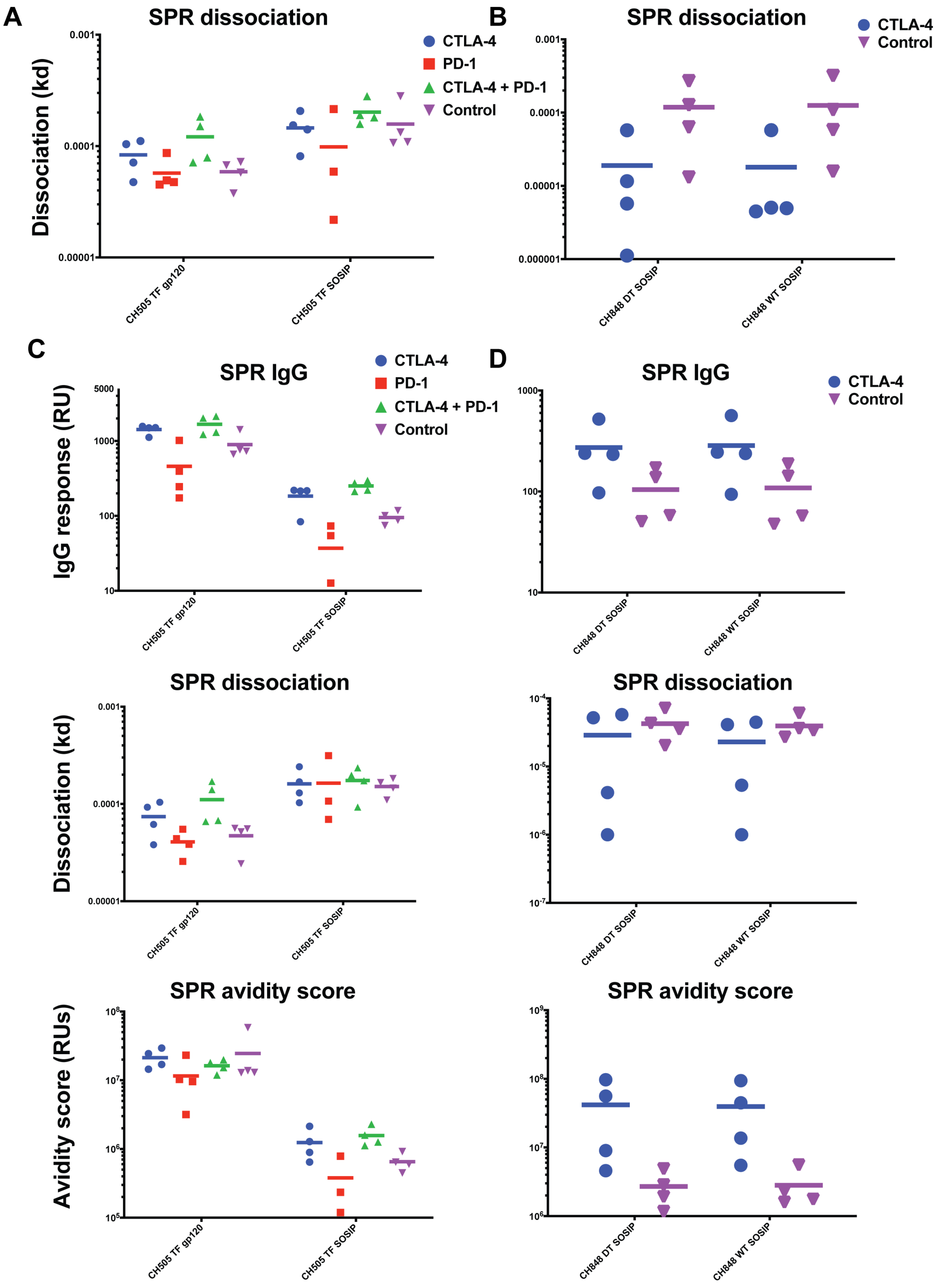

A
SPR dissociation
Dissociation (kd)
0.001
0.0001
0.00001
CTLA-4
PD-1
CTLA-4 + PD-1
Control
CH505 TF gp120
CH505 TF SOSIP
B
SPR dissociation
CTLA-4
Control
0.001
0.0001
0.00001
0.000001
CH848 DT SOSIP
CH848 WT SOSIP
C
SPR IgG
IgG response (RU)
5000
1000
100
10
CTLA-4
PD-1
CTLA-4 + PD-1
Control
CH505 TF gp120
CH505 TF SOSIP
D
SPR IgG
CTLA-4
Control
1000
100
10
CH848 DT SOSIP
CH848 WT SOSIP
SPR dissociation
Dissociation (kd)
0.001
0.0001
0.00001
CH505 TF gp120
CH505 TF SOSIP
SPR dissociation
10^{-4}
10^{-5}
10^{-6}
10^{-7}
CH848 DT SOSIP
CH848 WT SOSIP
SPR avidity score
Avidity score (RUs)
10^{8}
10^{7}
10^{6}
10^{5}
CH505 TF gp120
CH505 TF SOSIP
SPR avidity score
10^{9}
10^{8}
10^{7}
10^{6}
CH848 DT SOSIP
CH848 WT SOSIP

Supplementary Figure 2. Dissociation and replicate measurements of plasma IgG binding and avidity by SPR. **(A-B)** Dissociation measurements reported as kd values of purified plasma IgG measured by SPR for **(A)** Macaque study #1 to CH505 TF gp120 and CH505 TF SOSIP trimer proteins and **(B)** Macaque study #2 to CH848 DT and CH848 WT SOSIP trimer proteins. Each dot represents a single animal and are colored based on antibody treatment group (NHP study #1, CTLA-4, blue; PD-1, red; CTLA-4 + PD-1, green; Control CH65, purple; NHP study #2, CTLA-4, blue; Control CH65, purple). (**C-D)** Replicate experiment SPR measurments of plasma antibody IgG binding, dissociation and avidity for **(C)** Macaque study #1 to CH505 TF gp120 and CH505 TF SOSIP trimer proteins and **(D)** Macaque study #2 to CH848 DT and CH848 WT SOSIP trimer proteins. Each dot represents a single animal and are colored based on antibody treatment group (NHP study #1, CTLA-4, blue; PD-1, red; CTLA-4 + PD-1, green; Control CH65, purple; NHP study #2, CTLA-4, blue; Control CH65, purple). Source data are provided as a Source Data file.

A

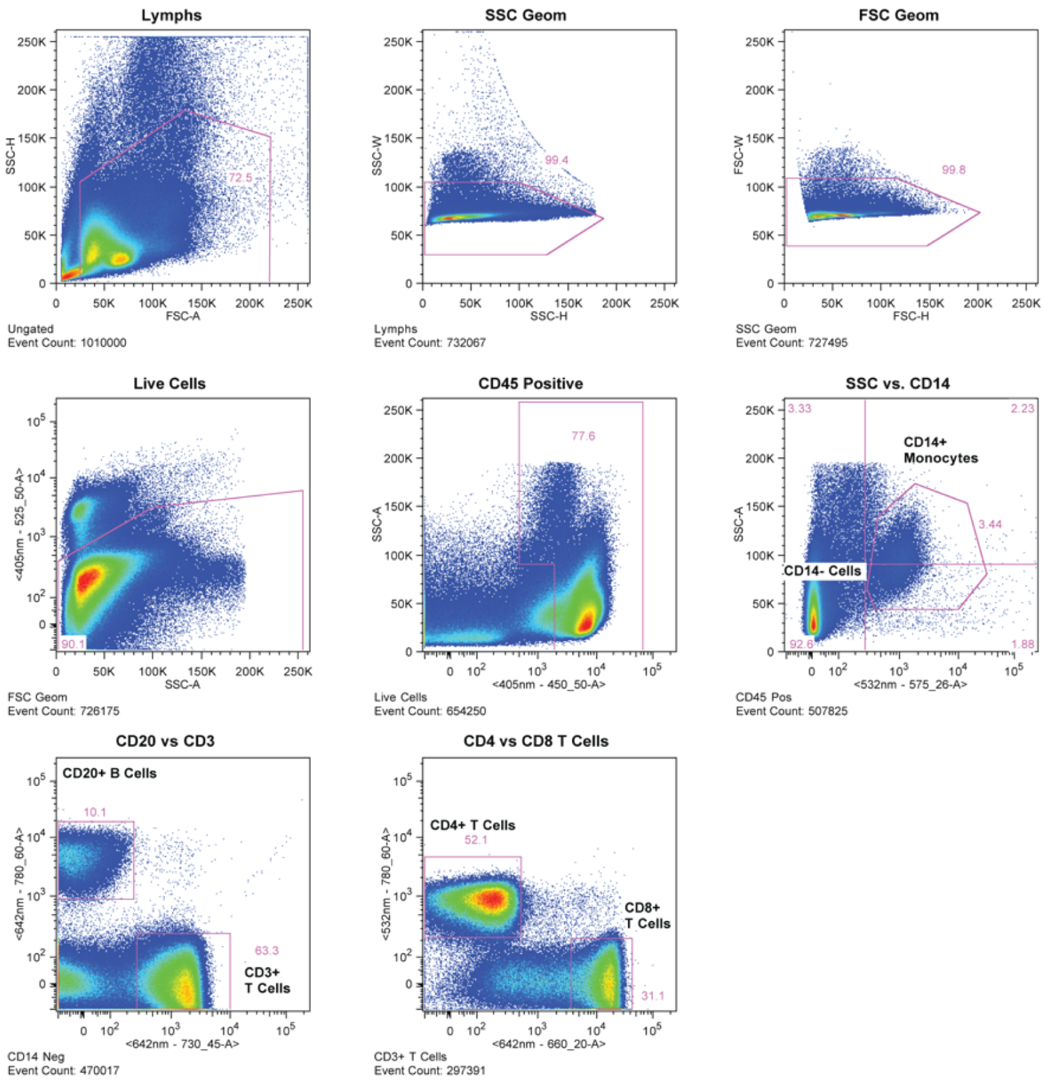

B

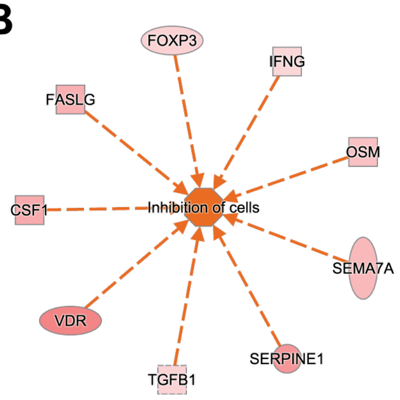

C

	CTLA-4		CTLA-4+PD-1	
Transcript	LOG FC	p.adj	LOG FC	p.adj
ZNF532	2.59	5.54E-06	2.34	0.002442
CSF1	2.07	0.01739778	2.29	0.00169646
FAM89A	1.12	0.03406864	1.23	0.0359972
DLL1	1.67	1.73E-05	1.68	0.04343894

Supplementary Figure 3. RNA-seq of macaque CD4+ T and B cell populations. **(A)** Representative flow plot outlining gating strategy of monkey B and T cells used for RNA-seq in Figure 3. **(B)** Igenuity (Qiagen) Pathway analysis of significantly changed genes in the PD-1 treated macaques revealed genes changed in the pathway inhibition of cells. **(C)** Transcripts that overlap between CTLA-4 and CTLA-4 + PD-1 B cells compared to control with log fold change and adjusted P value (t-test;Bonferoni adjusted).

Week 0	Control	CTLA-4 Ab	PD-1 Ab	CTLA-4 + PD1 Ab	Week 6	Control	CTLA-4 Ab	PD-1 Ab	CTLA-4 + PD1 Ab
WBC	10.423	10.063	9.918	9.738	WBC	12.043	10.823	6.865 *	12.38
RBC	5.9	5.763	6.023	5.723	RBC	5.345	5.36	5.378	5.27
MPV	9.625	10.375	9.75	11.15	MPV	10.475	10.825	9.825	11.05
MCV	71.425	75.3	69.725	70.725	MCV	73.3	77.225	70.8	71.875
MCH	20.7	21.925	19.825	20.225	MCH	20.675	21.8	19.7	20.125
HGB	12.2	12.625	11.925	11.575	HGB	11.075	11.675	10.575	10.6
HCT	42.1	43.4	41.925	40.55	HCT	39.125	41.375	37.95	37.875
%Lymph	43.4	38.55	41.225	40.1	%Lymph	30.325	39.3	41.85	35.125
%Neut	49.1	53.025	52.35	55.375	%Neut	62.35	50.075	51.05	60.175
%Mono	3.2	3	3.325	2.95	%Mono	2.85	3.075	3.025	2.725
%Luc	1.35	1.425	1.575	1.075	%Luc	0.925	1.55	1.2	1.45
%Eso	4.25	5.35	3.025	1.45	%Eso	4.45	7.475	4	1.875
%Baso	0.1	0.05	0.075	0.1	%Baso	0.075	0.1	0.075	0.125
PLT	360.25	353.75	363.5	287.75	PLT	347	368	376	261

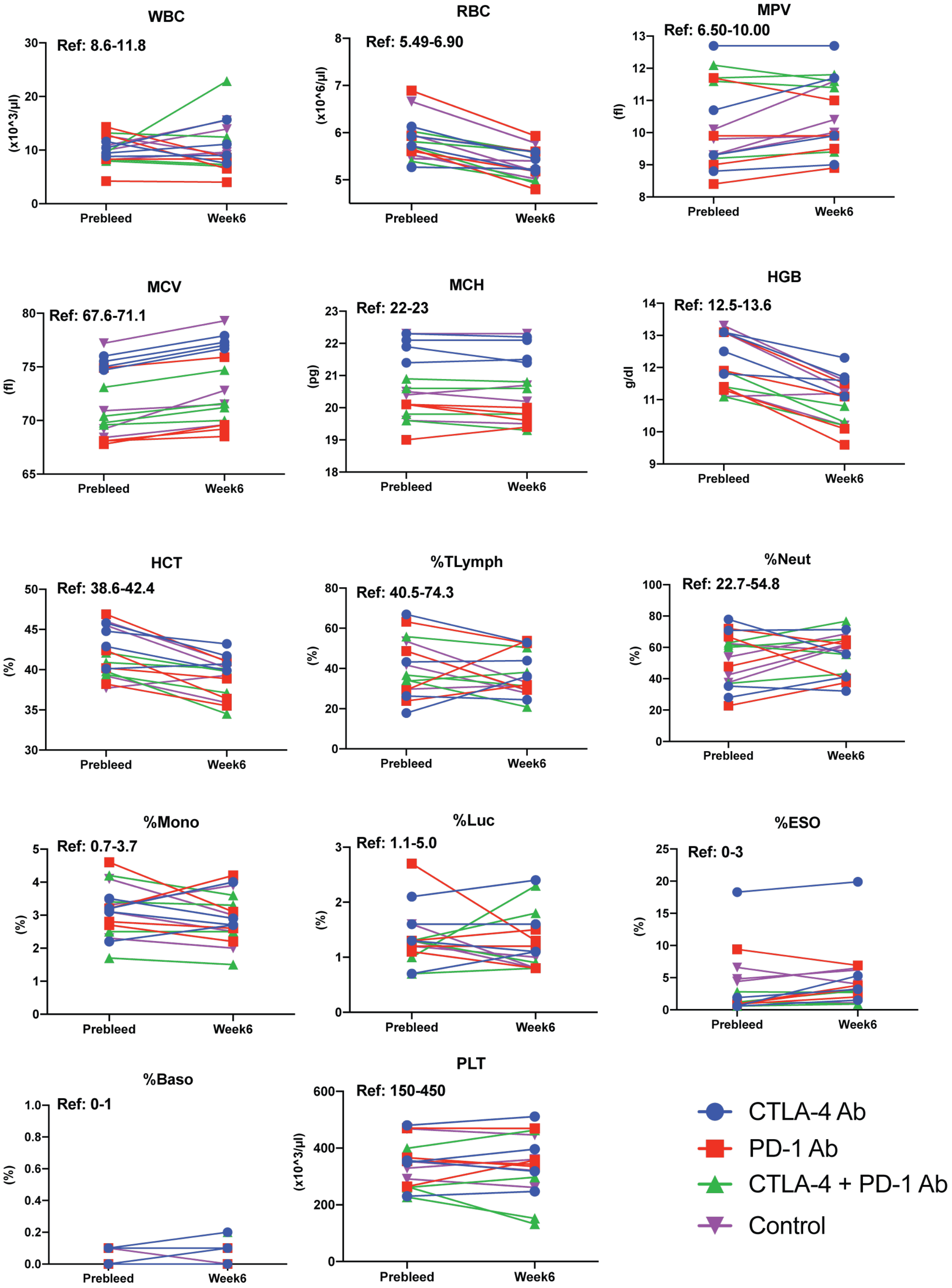

Supplementary Figure 4. Peripheral blood counts before immunization and antibody co-administration and after two immunizations and antibody coadministration (week 6) for all macaques in each group. Table shows group (n=4) averages for each measurement with macaque or human reference values shown. * P < 0.05 compared to control animals. Wilcoxon-Mann-Whitney.

Week 0	Control	CTLA-4 Ab	PD-1 Ab	CTLA-4 + PD1 Ab	Week 6	Control	CTLA-4 Ab	PD-1 Ab	CTLA-4 + PD1 Ab
ALT	40.75	30.75	48.5	52.75	ALT	63	47	55.75	52.25
AST	35	38.25	39.75	39	AST	46.75	45.75	38.25	39.5
AlkPhos	507.5	465.5	386.5	533.75	AlkPhos	342	286.75	260.75	348.25
Creatinine	0.55	0.55	0.675	0.6	Creatinine	0.625	0.55	0.65	0.625
CPK	531.5	451.751	351.25	1203.5	CPK	518	568.75	278	192
Albumin	3.95	3.9	4.075	3.8	Albumin	4.025	3.875	3.875	3.675
Globulin	2.8	2.875	3.125	2.875	Globulin	3.1	3.125	3.325	3.375
A/G	1.425	1.35	1.3	1.325	A/G	1.3	1.225	1.175	1.125
Bil	0.1	0.15	0.2	0.125	Bil	0.1	0.15	0.15	0.175
Bun	16	12.5	15.5	16	Bun	10.75	11	12.25	13.25
Protein	6.75	6.775	7.2	6.675	Protein	7.125	7	7.2	7.05
Glucose	55.25	52	46	56	Glucose	62.75	56.5	55.25	50.75
Calcium	9.775	9.925	9.75	9.825	Calcium	10.075	9.85	9.825	9.75
Chloride	109.5	109.5	108	109.25	Chloride	109	109.5	108.25	107.75
Cholesterol	85.25	99	110	92.25	Cholesterol	84	91.25	97.75	87.75
Phos	5.075	5.125	4.45	5.225	Phos	3.85	3.9	3.85	4.425
Potasium	3.65	3.625	3.6	3.75	Potasium	3.5	3.425	3.475	3.475
Sodium	144.5	144.75	143.5	144	Sodium	145.5	145.5	144.25	144.25

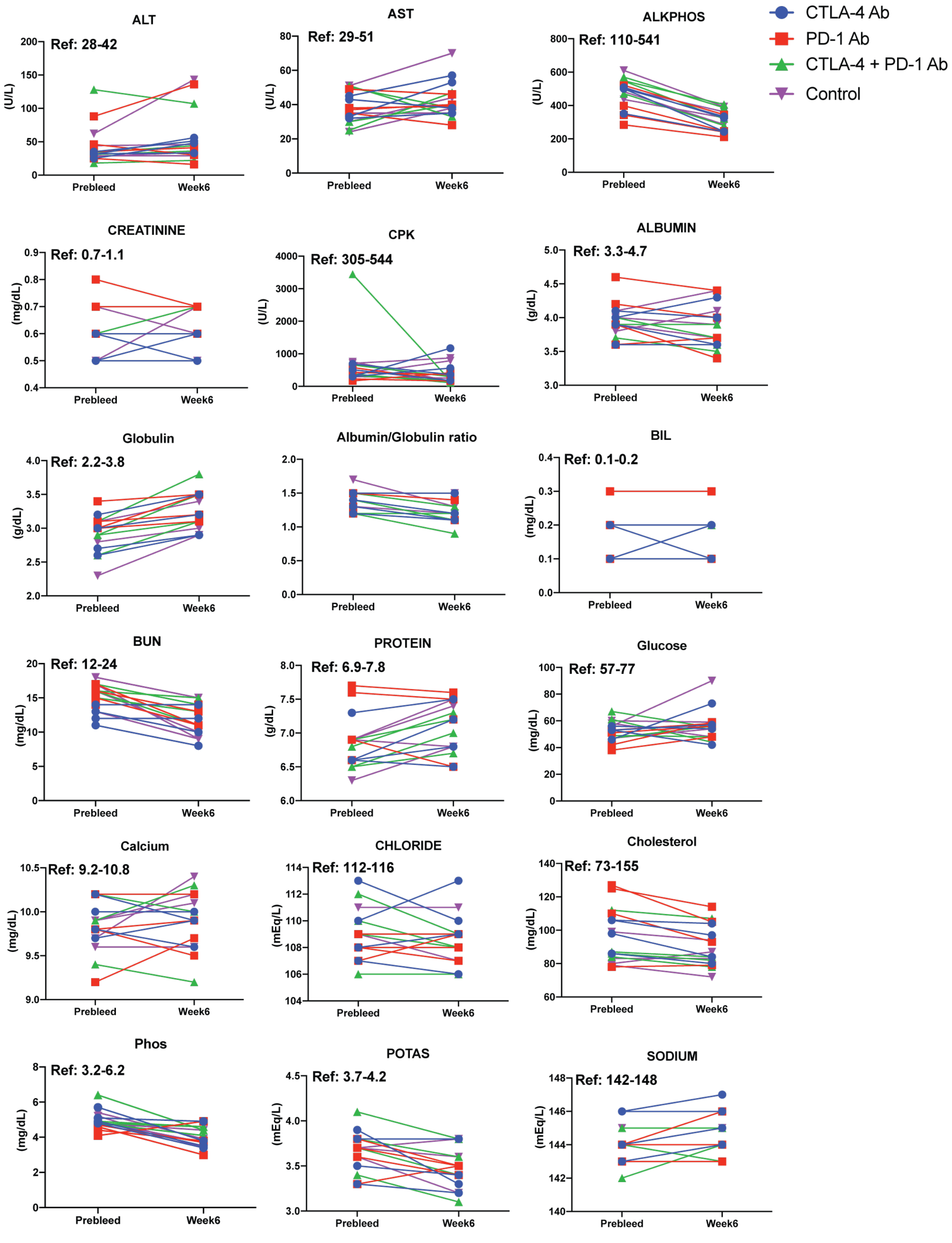

Supplementary Figure 5. Peripheral blood chemistries before immunization and antibody co-administration and after two immunizations and antibody coadministration (week 6) for all macaques in each group. Table shows group (n=4) averages for each measurement. Reference values for rhesus macaques shown.

A

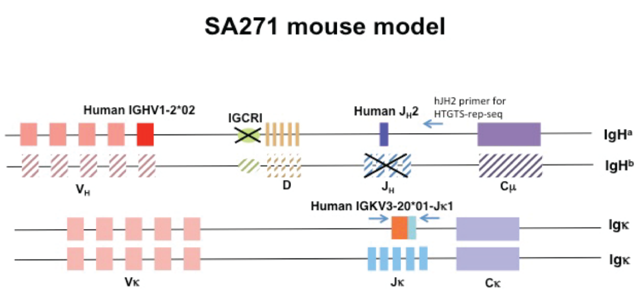

B

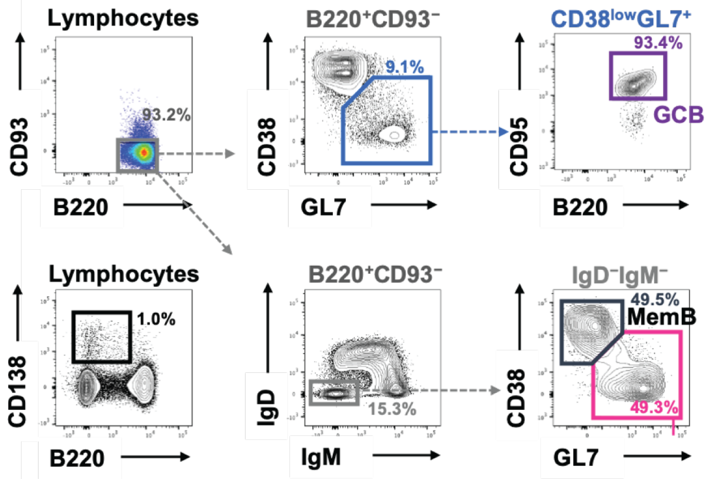

C

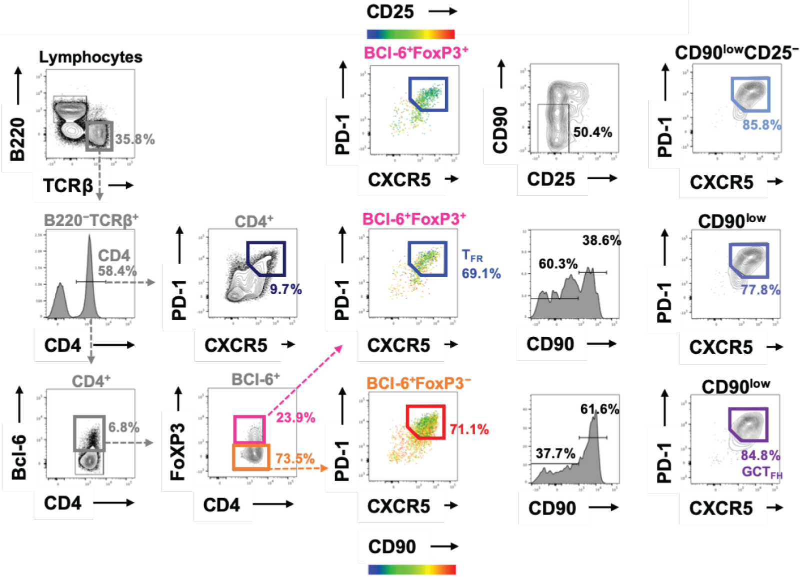

D

Group	Mouse	Productive Reads	Mouse Prod Reads	huVH1-2 Prod Reads	VH1-2 Prod (%)	VH1-2 Mut Freq			Median Mouse Mut Freq	Num Clusters	Clusters/10 K Reads	Entropy	Mouse CDRH3 Length		
						Q25	Median	Q75					Q25	Median	Q75
Control No Ab	V224	51505	36250	15255	29.6%	0.69%	2.08%	3.86%	3.42%	1481	408.6	3.698	33	36	39
	V225	16271	11982	4289	26.4%	1.04%	2.85%	4.51%	4.42%	262	218.7	3.611	33	36	39
Control IgG	V232	50837	34215	16622	32.7%	0.69%	2.70%	4.68%	3.51%	1860	543.6	3.670	33	36	42
	V233	46441	34874	11567	24.9%	0.35%	1.05%	2.78%	3.13%	2727	782.0	3.637	30	36	39
CTLA4	V220	55171	48047	7124	12.9%	0.69%	1.39%	2.43%	3.82%	3349	697.0	3.249	30	36	39
	V221	57401	49943	7458	13.0%	0.37%	1.39%	3.17%	3.40%	3716	744.0	3.031	30	36	39
	V222	44224	36392	7832	17.7%	0.00%	0.69%	1.74%	3.33%	1920	527.6	3.357	33	36	39
	V223	44631	36005	8626	19.3%	0.00%	0.69%	1.74%	3.19%	1961	544.6	3.415	33	36	39
OX40	V227	50978	32339	18639	36.6%	1.47%	2.78%	4.20%	3.86%	1340	414.4	3.605	33	36	42
	V228	6699	5790	909	13.6%	1.39%	2.43%	3.82%	4.51%	320	552.7	3.563	36	36	42
	V229	43653	37082	6571	15.1%	0.69%	2.08%	3.50%	3.47%	2235	602.7	3.442	33	36	39

E

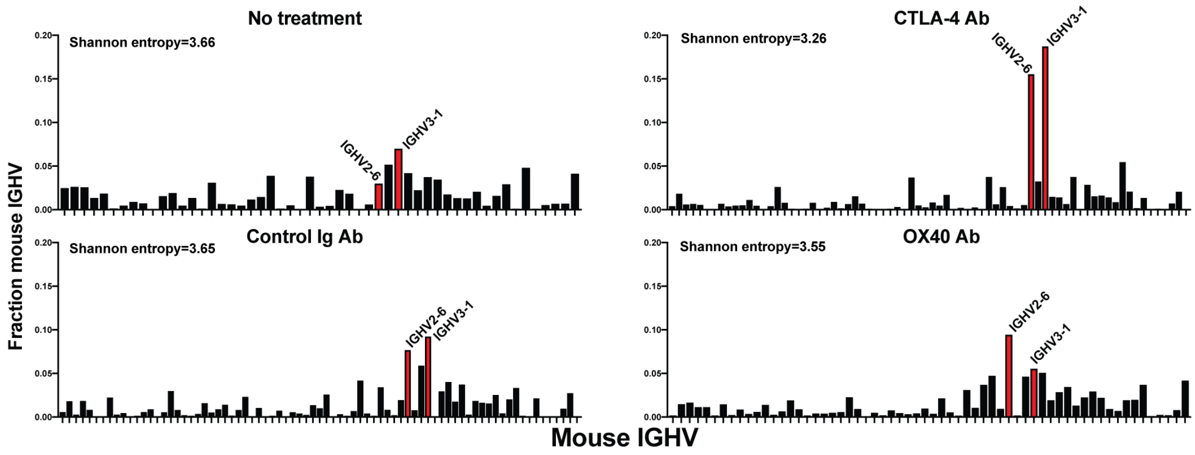

Supplementary Figure 6. VRC01 germline knock-in mouse design and sequencing (A) Schematic showing the design of the VRC01 germline knock-in mouse and location of primers for HTGTS-Rep-Seq. **(B-C)** Representative flow cytometry gating strategy to identify **(B)** germinal center and memory B cells in Figure 6A-6B and **(C)** T follicular helper and regulatory cells in Figure 6C-6D from mouse spleen cells. **(D)** Metrics of the HTGTS-Rep-Seq from splenic germinal center B cells for knock-in VH1-2 human gene sequences and endogenous mouse IGHV sequences. **(E)** Histogram of the frequency of mouse immunoglobulin IGHV gene segment usage in each group. Only genes detected in all animals in each group averaged and shown. Source data are provided as a Source Data file.
