# Supplementary material for: Immune checkpoint modulation enhances HIV-1 antibody induction
Source: Nat Commun. 2020 Feb 19;11:948. doi: 10.1038/s41467-020-14670-w (PMC7031230; doi:10.1038/s41467-020-14670-w)
Supplement: Supplementary file 3 — Reporting Summary [file 41467_2020_14670_MOESM3_ESM.pdf]

nature research

Corresponding author(s): Todd Bradley and Barton Haynes

Last updated by author(s): Jan 8, 2020

Reporting Summary

Nature Research wishes to improve the reproducibility of the work that we publish. This form provides structure for consistency and transparency in reporting. For further information on Nature Research policies, see Authors & Referees and the Editorial Policy Checklist .

Statistics

For all statistical analyses, confirm that the following items are present in the figure legend, table legend, main text, or Methods section.

n/a	Confirmed	
☐	☒	The exact sample size (*n*) for each experimental group/condition, given as a discrete number and unit of measurement
☐	☒	A statement on whether measurements were taken from distinct samples or whether the same sample was measured repeatedly
☐	☒	The statistical test(s) used AND whether they are one- or two-sided<br>*Only common tests should be described solely by name; describe more complex techniques in the Methods section.*
☐	☒	A description of all covariates tested
☐	☒	A description of any assumptions or corrections, such as tests of normality and adjustment for multiple comparisons
☐	☒	A full description of the statistical parameters including central tendency (e.g. means) or other basic estimates (e.g. regression coefficient) AND variation (e.g. standard deviation) or associated estimates of uncertainty (e.g. confidence intervals)
☐	☒	For null hypothesis testing, the test statistic (e.g. *F*, *t*, *r*) with confidence intervals, effect sizes, degrees of freedom and *P* value noted<br>*Give P values as exact values whenever suitable.*
☒	☐	For Bayesian analysis, information on the choice of priors and Markov chain Monte Carlo settings
☐	☒	For hierarchical and complex designs, identification of the appropriate level for tests and full reporting of outcomes
☐	☒	Estimates of effect sizes (e.g. Cohen's *d*, Pearson's *r*), indicating how they were calculated

Our web collection on statistics for biologists contains articles on many of the points above.

Software and code

Policy information about availability of computer code

Data collection	Softmax Pro v7 was used to collect ELISA data, Flojo and FacsDiva version 10 to collect flow cytometry data.
Data analysis	Flojo and FacsDiva version 10 was used to analyze cell frequencies for flow cytometry; Bulk population RNA-seq was analyzed using Trim galore version 0.4.5, STAR v2.6 for alignment, HTSeq v0.11.1 for gene counting, Deseq2 for differential expression analysis and plots; Single-cell RNA-seq used the Cellranger v2.1 pipeline and R package Seurat analysis and figures; Graphpad Prism 7 was used to generate graphs

For manuscripts utilizing custom algorithms or software that are central to the research but not yet described in published literature, software must be made available to editors/reviewers. We strongly encourage code deposition in a community repository (e.g. GitHub). See the Nature Research guidelines for submitting code & software for further information.

Data

Policy information about availability of data

All manuscripts must include a data availability statement. This statement should provide the following information, where applicable:

- Accession codes, unique identifiers, or web links for publicly available datasets
- A list of figures that have associated raw data
- A description of any restrictions on data availability

The bulk RNA-seq and single-cell RNA seq data is available in the short read archive (SRA) under accession number PRJNA556211 and will be released to public upon publication. Figures 3and 7 display this data.

Field-specific reporting

Please select the one below that is the best fit for your research. If you are not sure, read the appropriate sections before making your selection.

- [x] Life sciences
- [ ] Behavioural & social sciences
- [ ] Ecological, evolutionary & environmental sciences

For a reference copy of the document with all sections, see nature.com/documents/nr-reporting-summary-flat.pdf

Life sciences study design

All studies must disclose on these points even when the disclosure is negative.

Sample size	This work was exploratory in nature and the number of animals in experimental and control groups were selected to be able to detect statistically significant differences using a nonparametric test without correction using a rank based test.
Data exclusions	No data was excluded from the analysis
Replication	The effect of CTLA-4 on HIV antibody responses was replicated in a second NHP study as well as in VRC01 KI and wild-type mouse models.
Randomization	Nonhuman primates and mice were randomly assigned to experimental groups in all vaccine studies.
Blinding	Researchers performing the assays were not blinded to the experimental groups but samples were not organized by experimental group during the experimental protocols.

Reporting for specific materials, systems and methods

We require information from authors about some types of materials, experimental systems and methods used in many studies. Here, indicate whether each material, system or method listed is relevant to your study. If you are not sure if a list item applies to your research, read the appropriate section before selecting a response.

Materials & experimental systems

n/a	Involved in the study	
[ ]	[x]	Antibodies
[ ]	[x]	Eukaryotic cell lines
[x]	[ ]	Palaeontology
[ ]	[x]	Animals and other organisms
[x]	[ ]	Human research participants
[x]	[ ]	Clinical data

Methods

n/a	Involved in the study	
[ ]	[ ]	ChIP-seq
[ ]	[x]	Flow cytometry
[ ]	[ ]	MRI-based neuroimaging

Antibodies

Antibodies used

rat anti-mouse CD16/32 (2.4G2, BD, 1/200) and rat serum IgG (Sigma-Aldrich, 1/200) in FACS buffer for 30 minutes and stained with fluorochrome-conjugated antibodies specific for CD4 (GK1.5,BioLegend, 1/200), CD25 (PC61, BioLegend, 1/200), CD38 (90, BioLegend, 1/200), CD44 (IM7, BioLegend, 1/400), CD62L (MEL-14, BD, 1/200), CD69 (H1.2F3, BioLegend, 1/200), CD80 (16-10A1, BD, 1/100), CD138 (281-2, BD, 1/400), B220 (RA3-6B2, BioLegend, 1/200), CXCR5 (2G8, BD, 1/100), GL7 (GL7, BD, 1/200), I-A/I-E (M5/114.15.2, BD, 1/400), ICOS (C398.4A, BioLegend, 1/200), IgD (11-26c.2a, BD, 1/200), IgM (II/4, eBioscience, 1/200), PD-1 (29F.1A12, BioLegend, 1/200), PD-L2 (TY25, BioLegend, 1/100) and TCRβ (H57-597, BD, 1/200) at 4°C for 30 minutes. LIVE/DEAD Fixable Near-IR Dead Cell Stain Kit (Life Technologies, #L34976, 1/1000) was subsequently used to determine the viability of cells. After surface staining, cells were washed, fixed, permeablized and stained with antibodies specific for Bcl-6 (K112-91, BD, 1/100) and FoxP3 (MF23, BD, 1/200).

FITC anti-human CD45RA (clone MEM-56; Thermo Fisher #MA1-19570; 1:20 dilution for staining), PE-Dazzle594 anti-human CXCR3 (clone 1C6/CXCR3; BD Biosciences #562451; 1:20), PE-Cy5 anti-human CD69 (clone FN50; Biolegend #310908; 1:50), PE-Cy7 anti-human CXCR5 (clone MU5UBEE; Thermo Fisher #25-9185-42; 1:50), APC-Cy7 anti-human CD3 (clone SP34-2; BD Biosciences #557757; 1:50), BV421 anti-human PD-1 (clone EH12.2H7; Biolegend #329920; 1:50), BV570 anti-human CD8 (clone RPA-T8; Biolegend #301038; 1:50), BV650 anti-human CD25 (clone BC96; Biolegend #302634; 1:50), BV711 anti-human CD4 (clone OKT4; Biolegend #317440; 1:50), and BV785 anti-human CD20 (clone 2H7; Biolegend #302356; 1:50). Cells were then incubated with LIVE/DEADTM Fixable Aqua Dead Cell Stain (ThermoFisher #L34957; 1:1000) to allow exclusion of dead cells. Cells were then fixed and permeabilized using FoxP3 Transcription Factor Buffers (ThermoFisher), and then labeled for intracellular antigens with the following fluorochrome-antibody conjugates: PE anti-human Bcl-6 (clone K112-91; BD Biosciences #561522; 1:50), APC anti-human FoxP3 (clone PCH101; Thermo Fisher #17-4776-42; 1:20), and AF700 anti-human Ki-67 (clone B56; BD Biosciences #561277; 1:50)

Validation

Mouse and human reagents were validated for recognition of targets by the manufacture, but human reagents were confirmed

to cross-react with Cynomolgus or Rhesus macaque cells prior to study. Antibodies were also titrated from manufacture to determine optimal titers using blood and lymph nodes from unrelated study.

Eukaryotic cell lines

Policy information about cell lines

Cell line source(s)	Expi293 or 293F cells (ThermoFisher, catalog #R79007)
Authentication	Cell were purchased from ThermoFisher that stated authentication.
Mycoplasma contamination	Cell lines were tested negative for mycoplasma contamination.
Commonly misidentified lines (See ICLAC register)	None

Animals and other organisms

Policy information about studies involving animals; ARRIVE guidelines recommended for reporting animal research

Laboratory animals	Mouse Balb/c and Cynomolgus macaques female and male animals were utilized.
Wild animals	None
Field-collected samples	None
Ethics oversight	Mice were housed at Duke and Monkeys were housed at Bioqual, Rockville, MD. The animals were maintained in accordance with the National Institutes of Health and Duke University guidelines and all studies were approved by the appropriate Institutional Animal Care and Use Committee.

Note that full information on the approval of the study protocol must also be provided in the manuscript.

ChIP-seq

Data deposition

☐ Confirm that both raw and final processed data have been deposited in a public database such as GEO.

☐ Confirm that you have deposited or provided access to graph files (e.g. BED files) for the called peaks.

Data access links<br>*May remain private before publication.*	*For "Initial submission" or "Revised version" documents, provide reviewer access links. For your "Final submission" document, provide a link to the deposited data.*
Files in database submission	*Provide a list of all files available in the database submission.*
Genome browser session (e.g. UCSC)	*Provide a link to an anonymized genome browser session for "Initial submission" and "Revised version" documents only, to enable peer review. Write "no longer applicable" for "Final submission" documents.*

Methodology

Replicates	*Describe the experimental replicates, specifying number, type and replicate agreement.*
Sequencing depth	*Describe the sequencing depth for each experiment, providing the total number of reads, uniquely mapped reads, length of reads and whether they were paired- or single-end.*
Antibodies	*Describe the antibodies used for the ChIP-seq experiments; as applicable, provide supplier name, catalog number, clone name, and lot number.*
Peak calling parameters	*Specify the command line program and parameters used for read mapping and peak calling, including the ChIP, control and index files used.*
Data quality	*Describe the methods used to ensure data quality in full detail, including how many peaks are at FDR 5% and above 5-fold enrichment.*
Software	*Describe the software used to collect and analyze the ChIP-seq data. For custom code that has been deposited into a community repository, provide accession details.*

Flow Cytometry

Plots

Confirm that:

- [x] The axis labels state the marker and fluorochrome used (e.g. CD4-FITC).
- [x] The axis scales are clearly visible. Include numbers along axes only for bottom left plot of group (a 'group' is an analysis of identical markers).
- [x] All plots are contour plots with outliers or pseudocolor plots.
- [x] A numerical value for number of cells or percentage (with statistics) is provided.

Methodology

Sample preparation	mouse and monkey lymph nodes were homogenized into single-cell suspensions iand resuspended and stained with antibodies in LN cells were resuspended in PBS containing 0.5%<br>bovine serum albumin, 0.1% sodium azide and 1mM EDTA (FACS buffer).
Instrument	Data were acquired on a BD LSRII flow cytometer
Software	analyzed using FlowJo version 10
Cell population abundance	The cell populations study were abundant enough to detect, we did not sort cells to assay for post-sort purity.
Gating strategy	For all cells forward and side scatter was used to enrich for Lymphocytes and then a live/dead gate was used to select living cells.<br>1. For macaque B and T cells CD45+ cells were selected then CD3+ vs CD20+ was used where the CD20+ cells were B cells. The CD3 T cells were further enriched by CD4+ staining. Bcl-6 and ki67 used to identify GC B cells and CXCR5+ and FoxP3 used to identify Tregs (Foxpe+ and Tfh (Foxp3-).<br>2. For mouse B cells B220+ followed by GL7 to identify germinal center B or IgM/IgD negative to identify memory B cells<br>3. For mouse T cells B220- and TCRb+ to identify T cells and PD1+CXCR5+ to identify Tfh and CXCR5+ Foxp3+ to identify Tfreg cells.

- [x] Tick this box to confirm that a figure exemplifying the gating strategy is provided in the Supplementary Information.

Magnetic resonance imaging

Experimental design

Design type	*Indicate task or resting state; event-related or block design.*
Design specifications	*Specify the number of blocks, trials or experimental units per session and/or subject, and specify the length of each trial or block (if trials are blocked) and interval between trials.*
Behavioral performance measures	*State number and/or type of variables recorded (e.g. correct button press, response time) and what statistics were used to establish that the subjects were performing the task as expected (e.g. mean, range, and/or standard deviation across subjects).*

Acquisition

Imaging type(s)	*Specify: functional, structural, diffusion, perfusion.*
Field strength	*Specify in Tesla*
Sequence & imaging parameters	*Specify the pulse sequence type (gradient echo, spin echo, etc.), imaging type (EPI, spiral, etc.), field of view, matrix size, slice thickness, orientation and TE/TR/flip angle.*
Area of acquisition	*State whether a whole brain scan was used OR define the area of acquisition, describing how the region was determined.*

Diffusion MRI ☐ Used ☐ Not used

Preprocessing

Preprocessing software	*Provide detail on software version and revision number and on specific parameters (model/functions, brain extraction, segmentation, smoothing kernel size, etc.).*
Normalization	*If data were normalized/standardized, describe the approach(es): specify linear or non-linear and define image types used for transformation OR indicate that data were not normalized and explain rationale for lack of normalization.*
Normalization template	*Describe the template used for normalization/transformation, specifying subject space or group standardized space (e.g. original Talairach, MNI305, ICBM152) OR indicate that the data were not normalized.*

Noise and artifact removal

Describe your procedure(s) for artifact and structured noise removal, specifying motion parameters, tissue signals and physiological signals (heart rate, respiration).

Volume censoring

Define your software and/or method and criteria for volume censoring, and state the extent of such censoring.

Statistical modeling & inference

Model type and settings

Specify type (mass univariate, multivariate, RSA, predictive, etc.) and describe essential details of the model at the first and second levels (e.g. fixed, random or mixed effects; drift or auto-correlation).

Effect(s) tested

Define precise effect in terms of the task or stimulus conditions instead of psychological concepts and indicate whether ANOVA or factorial designs were used.

Specify type of analysis: ☐ Whole brain ☐ ROI-based ☐ Both

Statistic type for inference
(See Eklund et al. 2016)

Specify voxel-wise or cluster-wise and report all relevant parameters for cluster-wise methods.

Correction

Describe the type of correction and how it is obtained for multiple comparisons (e.g. FWE, FDR, permutation or Monte Carlo).

Models & analysis

n/a	Involved in the study
☒	☐ Functional and/or effective connectivity
☒	☐ Graph analysis
☒	☐ Multivariate modeling or predictive analysis
