# Supplementary material for: Immune checkpoint modulation enhances HIV-1 antibody induction
Source: Nat Commun. 2020 Feb 19;11:948. doi: 10.1038/s41467-020-14670-w (PMC7031230; doi:10.1038/s41467-020-14670-w)
Supplement: Supplementary file 4 — Description of Additional Supplementary Files [file 41467_2020_14670_MOESM4_ESM.pdf]

**Description of Additional Supplementary Files**

File Name: Supplementary Data 1
Description: Differentially expressed transcripts in CTLA-4 Ab groups compared to control in CD4+ T cells.

File Name: Supplementary Data 2
Description: Differentially expressed transcripts in PD-1 Ab groups compared to control in CD4+ T cells.

File Name: Supplementary Data 3
Description: Differentially expressed transcripts in CTLA-4 + PD-1 Ab groups compared to control in CD4+ T cells.

File Name: Supplementary Data 4
Description: Differentially expressed transcripts in CTLA-4 Ab groups compared to control in CD20+ B cells

File Name: Supplementary Data 5
Description: Differentially expressed transcripts in PD-1 Ab groups compared to control in CD20+ B cells.

File Name: Supplementary Data 6
Description: Differentially expressed transcripts in CTLA-4 + PD-1 Ab groups compared to control in CD20+ B cells.

File Name: Supplementary Data 7
Description: Differential expressed transcripts in CTLA-4 Ab compared to control groups cells.

File Name: Supplementary Data 8
Description: Differential expressed transcripts in OX40 Ab compared to control groups cells.

File Name: Supplementary Data 9
Description: Differentially expressed transcripts in B cell cluster in CTLA-4 Ab compared to control groups.

File Name: Supplementary Data 10
Description: Differentially expressed transcripts in B cell cluster in OX40 Ab compared to control groups.
